# Surdité brusque: étude rétrospective à propos de 36 cas

**DOI:** 10.11604/pamj.2014.19.399.3677

**Published:** 2014-12-22

**Authors:** Karim Nadour, Mehdi Chihani, Youssef Darouassi, Mliha Touati, Mountassir Moujahid, Haddou Ammar, Brahim Bouaity

**Affiliations:** 1 Service d'ORL et de Chirurgie Cervico-Faciale, Hôpital Militaire Avicenne, Marrakech, Maroc; 2 Service de Chirurgie Générale, Hôpital Militaire Avicenne, Marrakech, Maroc

**Keywords:** Surdité brusque, étiopathogénie, traitement, facteurs pronostiques, sudden hearing loss, etiopathogeny, treatment, prognostic factors

## Abstract

L'Objectif de cette étude est de rapporter notre expérience concernant la prise en charge des surdités brusques en soulignant la notion d'urgence, et en montrant les facteurs influant la probabilité de récupération. Nous rapportons une étude rétrospective concernant 36 patients colligés au service ORL de l'Hôpital Militaire Avicenne de Marrakech au Maroc, pendant 05 ans. Uniquement les surdités brusques unilatérales ont été incluses dans notre étude. Il s'agit de 21 oreilles droites et 15 gauches. Les données cliniques étaient recueillies par l'interrogatoire et l'examen clinique complet. L’évolution du déficit auditif a été évaluée à l'admission, toutes les 48 heures et après arrêt du traitement par audiométrie tonale liminaire. Tous nos patients ont bénéficié des potentiels évoqués auditifs du tronc cérébral, 09 d'entre eux d'une tomodensitométrie. Une IRM a été réalisée chez une seule patiente. Le protocole thérapeutique comprend des corticostéroïdes, vasodilatateurs. Seulement 16,6% des patients ont récupéré la totalité de la perte auditive initiale. Les potentiels évoqués auditifs (P.E.A) ont décelé un cas de neurinome de l'acoustique confirmé par l'imagerie.

## Introduction

La surdité brusque (SB) se définit comme étant une surdité d'apparition soudaine de type perceptive, habituellement unilatérale et d’étiologie inconnue, associée ou non à des acouphènes et / ou des vertiges. Le but de notre travail est de souligner le caractère d'urgence de la SB et de montrer que le délai de la mise en route de traitement, l’âge avancé du patient, la coexistence de vertiges et le type de la courbe audiométrique peuvent influer sur la probabilité de récupération. Dans 10à 20% des cas, la SB est le premier symptôme d'un neurinome de l'acoustique (NA). Celui-ci doit être systématiquement recherché après une SB même si la récupération est complète. L'examen actuel le plus fiable est l'IRM des oreilles. D'autres causes plus rares de SB doivent aussi être exclues.

## Méthodes

Il s'agit d'une étude rétrospective réalisée au service d'ORL de l'Hôpital Militaire Avicenne de Marrakech de janvier 2007 à décembre 2012. L'analyse a porté sur 36 cas de surdité brusque. Seules les surdités brusques unilatérales sont retenues. Il s'agit de 21 oreilles droites et 15 gauches. Les données cliniques étaient recueillies par l'interrogatoire et l'examen clinique complet. Le déficit auditif a été évalué à l'admission, il est quotidien et après arrêt de traitement par audiométrie tonale liminaire. Tous nos patients ont bénéficié des potentiels évoqués auditifs du tronc cérébral, 09 d'entre eux d'une tomodensitométrie millimétrique en coupe axiale et coronale. Une IRM a été réalisée chez une patiente en raison d'une récidive homolatérale de la surdité brusque. Le protocole thérapeutique a été basé sur la corticothérapie associée à des vasodilatateurs pendant huis jours.

## Résultats

**Figure 1:**
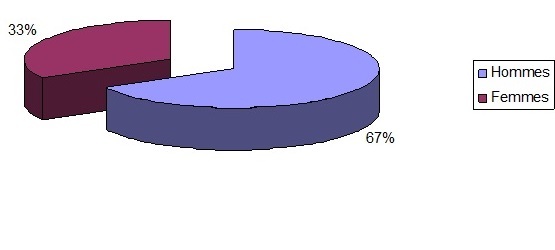
Répartition des sexes parmi les sujets de l’étude

Notre effectif est fait de 24 hommes et 12 femmes, âgés de 28 à 52 ans (Figure 1), l’âge moyen est de 36 ans expliquant la jeunesse de nos patients justifiée par un recrutement concernant une population militaire jeune. Le délai moyen de consultation de nos patients était de 19 jours. Ce retard de consultation est expliqué d'une part par l’éloignement du lieu de travail du patient par rapport à la formation médicale spécialisée et d'autre part par la sous-estimation du degré d'urgence par le patient et par son médecin de corps. Les antécédents et les signes associés sont résumés dans le Tableau 1.

**Tableau 1:** Résumé des antécédents pathologiques de nos patients, et les signes associés à la surdité

Antécédants pathologiques	Passé otitique	00 cas
	tabac	12 cas
	Contraceptifs oraux	06 cas
	HTA + diabète	06 cas
	dyslipidémie	03 cas
	Arthrose cervicale	02 cas
Signes associés	Sensation vertigineuse	03 cas
	acouphènes	02 cas
	Acouphènes et vertige	05 cas

**Figure 2:**
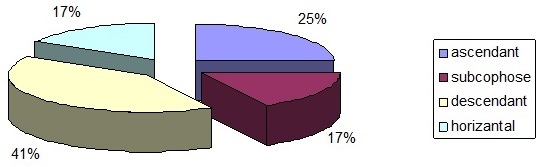
Distribution en pourcentage des différents types de courbe rencontrés

Le bilan électrophysiologique a été basé essentiellement sur l'audiométrie tonale liminaire (ATL) et les potentiels évoqués auditif du tronc cérébral (PEA-TC). L'ATL a permis de confirmer et de quantifier la surdité, ainsi nous avons noté une surdité de perception dépassant 35 dB chez 12 patients (09 hommes et 03 femmes), de 50 dB chez 18 patients (12 hommes et 06 femmes) et une subcophose chez 06 patients (03 hommes et 03 femmes). Le profil audiométrique a montré une courbe ascendante dans 09 cas (25%), descendante dans 15 cas (41%), horizontale dans 06 cas (17%) et subcophose dans O6 cas (17%) (Figure 2).

**Figure 3:**
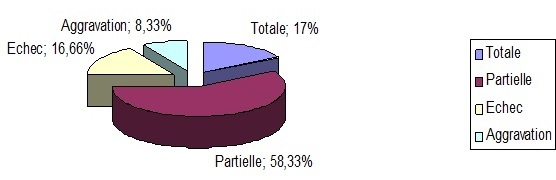
Pourcentage de récupération pour 36 malades

Les PEA-TC étaient demandés chez tous nos malades et montraient des tracés de type endocochléaire dans 27 cas, plat dans 08 cas et un cas de surdité retro cochléaire avec allongement de l'espace I-V justifiant un bilan radiologique tomodensitométrique complémentaire qui a pu déceler un NA de petite taille. La tomodensitométrie des conduits auditifs internes et de la fosse cérébrale postérieure a été demandée chez 09 personnes. Elle est normale dans 08 cas et a objectivé un petit NA dans l'autre cas. L'IRM n'a été faite que chez un patient. Sur le plan thérapeutique, nos patients sont hospitalisés à leur admission pour instaurer le traitement le plus précocement possible, avec suppression de tabac chez 12 hommes et de contraception orale chez 06 femmes, Des perfusions de vasodilatateurs type Piracetam à raison de 12g/j, et de corticoïdes type Methylprednisolone: 1mg/Kg/j ont été prescrites pendant une semaine relayé par un traitement per os de Piracetam pendant 4 semaines. Le seul NA a bénéficié d'un traitement neurochirurgical par voie rétro-sigmoïde. Le contrôle audiométrique quotidien a montré une récupération presque totale chez 06 patients (16,66%), une récupération de moins de 20 dB chez 21 patients (58,33%), une absence de récupération chez 06 patients (16,66%) et aggravation à type de cophose chez 03cas (8,33%) dont le patient opéré pour NA (Figure 3).

A long terme, nous avons noté une amélioration lentement progressive chez 15 patients, avec récidive homolatérale dans un cas bénéficiant d'une I.R.M ne montrant pas d'anomalies.

## Discussion

La SB constitue une entité nosologique authentique et idiopathique. A l’étude épidémiologique, il existe deux fourches d’âge de prévalence de la SB: entre 30 et 40 ans et entre 55et 60ans [1]. Les deux sexes sont atteints de façon égale. Le délai de consultation est en moyenne 04 jours [2], dans notre série il est de 19 jours. Les formes bilatérales représentent 08% [1]. Dans notre série, nous avons éliminé toutes les formes bilatérales de notre étude. Aux Etats- Unis 4000 nouveaux cas de surdité brusque sont notés par an [2, 3].

Sur le plan clinique, l'interrogatoire précise que c'est une surdité d'apparition brutale ou rapidement progressive, isolée ou associée à des acouphènes, diplacousie, et/ou vertiges. Il recherche des antécédents otologiques (chirurgie de la surdité, barotraumatisme), généraux (cardiopathies, hémopathies, diabète, maladies infectieuses) et une notion de prise médicamenteuse potentiellement ototoxique [3]. L'examen otoscopique est normal et l'acoumétrie montre que c'est une surdité de perception. Un examen vestibulaire est réalisé avec notamment la recherche d'un nystagmus spontané ou positionne. L'examen neurologique est négatif. L'ATL confirme cette surdité de perception d'au moins 30 dB sur trois fréquences consécutives, en précisant le degré et le type [4].

Un bilan biologique est demandé devant éliminer une affection susceptible de se révéler par une surdité brusque: numération-formule sanguine, vitesse de sédimentation, C reactive protein, bilan ionique et lipidique sanguin. Sérologie syphilitique; sérologie virale et bilan immunologique [5].

L'imagerie consiste en une IRM qui devrait être aujourd'hui systématique, permettant surtout d’éliminer un processus tumoral des conduits auditifs internes, des angles pontocérébelleux ou des structures nerveuses de la fosse postérieure; de rechercher des signes en faveur d'un accident ischémique dans le territoire de l'artère cérébelleuse antéro-inférieure et aider à poser l'indication d'une angiographie vertébrale conventionnelle pour le diagnostic de dissection vertébrobasilaire [6]. Les surdités brusques sont par définition idiopathiques, mais un certain nombre d'hypothèses pathogéniques permettent d'expliquer l’étiopathogénie de ces surdités et d'orienter le traitement. L'origine virale, essentiellement le virus du zona et ourlien sont responsables d’œdème des cellules endothéliales capillaires, d'une hémagglutination et d'une hypercoagulabilité sanguine [3, 7]. L'origine vasculaire est fortement suspectée [1, 2, 8, 9], parce que la vascularisation cochléaire est de type terminal et que la cochlée est particulièrement sensible à l'anoxie. Les étiologies vasculaires évoquées sont: L'athérosclérose avec ses facteurs de risque dont le tabac [5, 10], l'hémorragie intra-labirynthique, l'hypertension artérielle, la toxicité de la glutamine, l'augmentation de la viscosité sanguine [4, 6, 7, 11, 12] et les spasmes vasculaires. Les fistules et les ruptures du labyrinthe membraneux post -traumatique, l'infection bactérienne, l'ototoxicité des aminosides. Les affections neurologiques dégénératives (sclérose en plaque) et le NA peuvent se révéler par SB [1, 13]. L'origine auto-immune ou la syphilis [8, 14, 15] pourraient exister et expliquer des surdités brutales surtout lorsqu'elles sont bilatérales.

Le traitement des SB a pour but essentiel de restituer l'audition et d’éviter l'aggravation des lésions. Le repos au lit est recommandé et Plusieurs moyens thérapeutiques sont employés en mono ou plurithérapie selon les auteurs [8, 16]. Ces procédés ont pour objectifs d'augmenter le débit sanguin et la lutte contre l'anoxie de l'organe de corti par l'amélioration de la rhéologie sanguine: les vasodilatateurs, l'hémodilution [10, 12, 13]. Les anticoagulants, les antiagrégants plaquettaires [9, 10], l'inhalation de carbogène, l'oxygénothérapie hyperbare [9, 11]. La lutte contre l'inflammation par corticothérapie, systémique [12] et intra tympanique [13]; le traitement à visée pressionnelle, les antiviraux [14]. La vitaminothérapie (vitamine E) comme antioxydant et récemment le traitement par le magnésium, ont été essayés par plusieurs auteurs. Ces moyens thérapeutiques sont de valeurs inégales, c'est pourquoi les protocoles et leurs résultats sont tirés de l'expérience de plusieurs auteurs différents [15].

Il y a donc peu d'arguments pour retenir un protocole plutôt qu'un autre et les thérapeutiques sont utilisées diversement selon les écoles. Le rapport de la Société française d'ORL 2002 donne le protocole de Stennert utilisé dans les paralysies faciales, mais souligne qu'aucun traitement n'a fait la preuve de son efficacité réelle chez l'homme [16].

Méthylprednisolone: j1 – j2, intraveineuse lente; si le poids est inferieur à 70 kg: 100 mg deux fois par jour; si >70 kg: 120 mg 2 fois jour; j3 – j4: 80 mg 2fois /j; j5 – j6: 100 mg/j; j7: 75 mg/j. Pentoxifylline 400: 2 ampoules/j à j1 et j2; 3 ampoules/j de j3 à j7. Puis en ambulatoire: méthylprednisolone 16 mg ou prednisolone 20 mg: 4 comprimés/j à doses décroissantes jusqu’à j18 par paliers de 2 j; pentoxifylline 400: 3 comprimés/j [16]. L'hémodilution, les solutions hypertoniques, l'oxygène hyperbare et l'inhalation de carbogène sont utilisés de façon variable selon les centres [3, 16]. L’évolution des surdités brusques peut être spontanément résolutive vers la guérison et surtout chez les sujets jeunes de 15 à 40 ans. Elle est péjorative si la perte auditive est sévère (cophoses, subcophoses), le type descendant de la courbe audiométrique, l’âge avancé du malade et le retard de prise en charge thérapeutique (cas de notre petite série malgré la jeunesse de nos patients) ainsi qu’à l'association à d'autres signes notamment des vertiges [3, 16].

## Conclusion

La surdité brusque est une urgence médicale nécessitant une prise en charge rapide, globale et adaptée des patients atteints. Elle constitue une entité clinique bien individualisée même en l'absence d'un consensus de définition.. Toutes les modalités thérapeutiques actuelles tendent à augmenter la concentration d'oxygène dans les liquides endolymphatiques pour interrompre le processus anoxique et arrêter la libération d'anions ototoxiques et le glutamate au niveau de la synapse et donc arrêter l'extension des lésions. Le bilan de cette surdité doit comporter systématiquement la réalisation des PEA-TC dont l'altération est synonyme de la demande d'une IRM (qui devrait être actuellement systématique) à la recherche d'une tumeur de l'angle ponto-cérébelleux dont le NA ou une sclérose en plaque.
